# Beyond endogeneity in analyses of public opinion: Evaluations of healthcare by the foreign born across 24 European countries

**DOI:** 10.1371/journal.pone.0233835

**Published:** 2020-06-01

**Authors:** Simone M. Schneider

**Affiliations:** Max Planck Institute for Social Law and Social Policy, Munich, Germany; Augusta University, UNITED STATES

## Abstract

To address the problem of endogeneity in public opinion research, this study examines the opinions of healthcare held by the foreign born, i.e. those not socialized in the system they are asked to evaluate. It (a) explores the degree to which the healthcare ratings of the foreign born depend on the country’s institutional healthcare setting; (b) stresses the importance of referential standards and the significance of knowledge and previous experiences of healthcare services in the country of origin; and (c) investigates differences in healthcare ratings with the length of time foreign born spent in the destination country. This study uses data from the seven rounds of the European Social Survey (2002–2014) and applies multilevel modelling techniques. Results show the institutional characteristics of healthcare services in the country of residence are associated with healthcare evaluations of the foreign born, in particular if these services are compared to those in the country of origin: the better healthcare institutions perform relative to those in the country of origin, the higher the healthcare ratings. Although comparisons with the country of origin seem relevant to all foreign born, they are sometimes more important to recent arrivals. This study suggests knowledge and experience of different healthcare institutions change perspectives and evaluations of healthcare. This finding enriches the discussion of the effects of socialisation and adaptation processes in the formation of public opinion.

## Introduction

Given sweeping demographic changes, technological developments, and budgetary constraints, policy makers in many European countries are being forced to re-think healthcare [1]. The bottom line may be cost, but decision makers may also consider whether healthcare systems meet the needs and interests of the general public and if they comply with their norms and values. To this end, research on the public’s opinion of healthcare may offer valuable insights [2,3]. The general public’s healthcare ratings–users and non-users alike–provide a broad and composite assessment of a healthcare system [4] and express the public’s support for it [5,6]. Scholars say these ratings help to assess the ‘subjective’ performance of healthcare systems [7,8], and they can–at times–drive policy change [9]. Indeed, studies show that healthcare ratings follow a coherent cognitive reasoning and that they vary systematically between individuals and social groups. Values (e.g. egalitarian norms), as well as interests and needs tied to demographic (e.g. age and gender) and socioeconomic characteristics (e.g. income, education, status of employment), determine how individuals evaluate healthcare [10–14]. Their experiences with healthcare services [15,16], confidence in finding affordable and effective care [17], and perceptions of efficiency and equality of health treatment [18] also influence their opinions.

Cross-country comparative analyses of the public’s opinion of healthcare are particularly informative to policy research. They can show the extent to which the public’s opinion of healthcare is shaped by the institutional framework of the healthcare system [9,16,19]. Research in this direction can clarify which policies and institutional settings are performing better with respect to meeting the needs and interests of the general public [8]. Indeed, studies show that some institutional factors are related to healthcare ratings. For example, countries with higher total health expenditure tend to report higher healthcare ratings [20], as do countries with higher public health expenditure [12]. Interestingly, out-of-pocket payments are not always related to healthcare evaluations [20,21]. Research also suggests the density of primary care services can have a positive and significant effect [11,20,21], but the density of specialists and the number of hospital beds may not [20]. Surprisingly, and despite growing research efforts, empirical studies find no empirical association between healthcare evaluations and institutions that regulate access to medical care [11,20]–factors that are closely related to the individual’s healthcare seeking behaviour and directly shape his or her experiences [22].

As appealing as this cross-comparative research strategy might be, it also bears potential risks and may lead to mistaken conclusions. Current debates about path-dependency and policy feedback point to the ‘problem of endogeneity’: public opinion may not only influence social policy; social policy may ‘feedback’ and influence the public’s opinion as well [23]. More specifically, neo-institutionalist theory argues that public institutions endow meaning and convey normative values [24]. Preferences and expectations are formed within institutional structures and naturally develop from adaptation and socialization processes [25–29]. Familiarity with a particular system, paired with the unawareness that institutions can be arranged differently, may legitimate the status quo [30]. Consequently, institutions may be taken for granted and left unquestioned [31]. If this is the case, the findings of cross-country comparative research on the public’s opinion of social policies may not properly reflect variations in the actual performance of public institutions or changes in social policy. This could explain why the public’s evaluation of healthcare does not always vary with the institutional characteristics of the healthcare system itself.

This study contributes to research on the institutional determinants of healthcare evaluations in Europe and potential feedback effects, i.e. the role of socialization and adaptation processes, in particular, experience and knowledge of alternative institutional arrangements, in shaping responses to healthcare services. It addresses the problem of endogeneity in public opinion research within the context of health policy by examining healthcare evaluations *from the perspective of the foreign born*. The foreign born are an interesting (and rapidly growing) study population: they have lived in different institutional settings and, thus, evaluate public institutions based on their experience and knowledge of alternative institutional arrangements [32,33]. Therefore, socialisation and adaptation processes within a particular institutional system and the influence of institutional structures of the host country vs those of the country of origin can be studied simultaneously [34]. At the same time, opinions of the foreign born are unlikely to influence social policy, as the foreign born are seldomly perceived as a politically salient group with common healthcare needs and interests. This allows scholars to avoid problems of causality, i.e. the dual relationship between social policy and public opinion, but to focus on the various institutional effects on public opinion.

This study uses data of the seven rounds of the European Social Survey (2002–2014) and examines healthcare evaluations of the foreign born in Europe, i.e. persons originally from Europe now residing in a European country in which they were not born. Specifically, it explores the degree to which their evaluations of healthcare services depend on the performance of the host country’s healthcare system; it looks for referential standards expressed in experiences with healthcare services in the country of origin; and it investigates adaptation processes, i.e. changes in healthcare evaluations with the length of time spent in the host society. Results provide a deeper understanding of how European healthcare systems are perceived by those who have not been socialized in the system they are asked to evaluate and who are likely to apply different frames of reference, given their awareness and experience of alternative healthcare arrangements.

### Theoretical framework

#### The health system effect: Institutions matter!

The empirical study of how institutional forces affect healthcare evaluations requires an analytical framework capable of guiding the selection of appropriate indicators and assessing institution-specific effects. Typically, welfare state regimes show little relationship to health systems and are of little use to health policy research [35]. Even healthcare typologies that group countries into analytically meaningful categories [36,37] are often too broad to study the impact of specific healthcare institutions [20]. Particularly useful for the empirical study of institutional forces on healthcare evaluations is the classification by Wendt and colleagues of three distinct institutional dimensions of the ‘production process’ of healthcare services [2,8,9,11]: (i) ‘monetary input’ accounts for the financing of healthcare services; (ii) ‘real input’ reflects the supply of human resources in terms of both healthcare facilities and personnel; and (iii) ‘institutional set-up’ regulates access to healthcare services. Although these are interrelated, the impact of each on public opinion can be studied separately.

Generally, individuals are expected to rate health systems more positively when the system better meets their needs, interests and values; that is, with larger amounts of financial resources flowing into the system and smaller amounts of out-of-pocket expenditures, higher provision of health services and personnel, and greater choice accessing healthcare services. In this, the foreign born should be no different from the general population. However, empirical studies only partly support these expectations for the general population. As stated above, a robust and positive relationship could only be established for healthcare evaluations and the amount of total and public health expenditure as well as the density of primary care services. Surprisingly, the amount of out-of-pocket payments, the density of specialists, the number of hospital beds, and access regulations have no robust effects on the public’s opinion of healthcare [11,12,20,21]. These results could be hampered by socialization and adaptation effects within specific institutional settings. If this is the case, the expected associations between healthcare institutions and the opinion towards healthcare should be reflected more clearly in the empirical results of the foreign born population. Thus, the first hypothesis states:

*H1a: The foreign born will evaluate healthcare services in the country of residence more positively when these feature larger amounts of financial (public) resources and fewer out-of-pocket expenditures, greater provision of healthcare services and personnel, and more choice accessing healthcare services*.

Of course, individuals do not perceive healthcare systems in a social vacuum [38]. Opinions of public institutions are also informed by informal networks and the common evaluation and appreciation of these institutions by the larger public. While research on the relevance of these ‘soft’ institutional factors is often trapped in problems of endogeneity, this study’s research design discriminates between and links two populations, i.e. the opinions of the native majority and the opinions of the foreign born. The healthcare opinions of the native born are expected to shape the healthcare evaluations of the foreign born. Therefore, the institutional hypothesis (H1a) is extended by a ‘soft’ institutional factor, the natives’ opinion of healthcare, and states:

*H1b: The foreign born will be more positive about the healthcare services in the country of residence when the native born are more positive*.

#### The reference frame effect: Knowledge and experience of alternatives matter!

Social comparisons are a central psychological process crucial for individuals to evaluate their personal abilities and to form and re-evaluate their opinions [39]. As the foreign born have experienced different institutional settings, they are likely to perceive and interpret information on public institutions in the host country differently. Hence, the institutional characteristics of the host country will be perceived and evaluated in the context of experiences in the country of origin.

Migration research supports this expectation and shows migrants apply a ‘dual frame of reference’ when evaluating institutions in the host society, considering both their characteristics and those of institutions in their country of origin. For example, Röder and Mühlau find differences in political trust between first-generation immigrants and the native born are fully explained by comparison processes: the better the institutional performance in the host country compared to the country of origin, the higher the institutional trust of these immigrants [34]. In a study of immigrants in Western Europe, Dinesen finds the level of general trust is influenced by both cultural heritage and institutional aspects of the host society: the higher the level of trust in the country of origin and the lower the level of corruption in the destination country, the higher the level of general trust [32].

Against this background, it seems likely that the foreign born will judge health services in their country of residence based on their previous experiences in their country of origin. Therefore, this study expects to find systematic variations in healthcare evaluations between different groups of foreign born and assumes these variations can be explained by the institutional performance of healthcare systems in the country of residence relative to the country of origin. This leads to the following two hypotheses:

*H2a: The foreign born will be more positive about healthcare services in the country of residence when there are larger amounts of financial resources and fewer out-of-pocket expenditures, greater provision of healthcare services and personnel, and more choice accessing healthcare services in the country of residence than in the country of origin*.
*H2b: The foreign born will be more positive about healthcare services in the country of residence when the evaluation of healthcare services by the native born is more positive than healthcare evaluations in the country of origin*.

#### The newcomer effect: Length of residency matters!

If attitudes towards public institutions, in this case healthcare services, can be explained by socialisation and adaptation processes, the length of time a foreign-born person resides in a country will affect how he or she perceives its public institutions. In line with migration research [34,40–42], this study expects the opinions of recent arrivals compared to those of earlier arrivals (i) will be more positive about the host country institutions because of an initial optimism that motivated migration in the first place, and (ii) will depend more strongly on comparisons with institutions in the country of origin as their experiences are more recent. The following two hypotheses express these expectations:

*H3a: Recent arrivals will be more positive about healthcare services in the country of residence than those who have resided in the country for a longer period*.
*H3b: The more recently the foreign born have entered the host country, the more important the characteristics of the country of origin will be in their evaluations of healthcare services in the country of residence*.

## Methods

### Data

The empirical analysis is based on data of the European Social Survey (ESS). The ESS is a high quality, cross-comparative data set with biannual information representative of the resident national population living in private households aged 15 and above. It follows a repeated cross-sectional design, and respondents are selected using strict probability sampling. Data are collected via face-to-face interviews. All data are publicly available for not-for-profit purposes at the European Social Survey website (visit the following link: www.europeansocialsurvey.org) and comply with highest ethical standards. Questionnaires are developed and pretested by international teams and discussed and approved by an international consortium. The data, instruments, field work and quality assessments are well documented on the website. Data are anonymous so that individual survey participants cannot be identified. It is noteworthy that the ESS does not apply a specific sampling scheme for the foreign born and seeks to interview residents regardless of their nationality, citizenship or language. Questionnaires are prepared for each language used by at least five per cent of the population in any given country. This paper uses the first seven rounds of the ESS and covers 24 European countries over 12 years (2002–2014). As a country’s participation in the ESS is voluntary, information on some European countries is fully missing or available for specific time points only.

### Study population

The study population, the foreign born, are defined as persons originally from Europe now residing in a European country in which they were not born. I restrict the sample to the foreign born from Europe to reduce cultural heterogeneity in the interpretation and evaluation of European healthcare services. This also enables me to test for ‘soft’ institutional indicators outlined in Hypothesis 1b and 2b, such as opinions of healthcare services in the country of residence and country of origin which are available in the ESS.

Further, I restrict the sample to the foreign born who entered the destination country at the age of 18 and above. This ensures respondents entered as adults and experienced other healthcare services for a considerable time. Please note that for the first four rounds of survey, the age of immigration to the country of residence had to be estimated using categorical information (less than 1 year, 1 to 5 years, 6 to 10 years, 11 to 20 years, and more than 20 years) on the length of time the foreign-born person has resided in the country of residence. Furthermore, and because there is insufficient information on the time of emigration from the country of birth, it remains uncertain whether respondents actually lived there until entering the country of residence; it is possible that they went elsewhere first.

I also exclude groups of foreign born with fewer than three respondents to avoid potential biases in the estimation process. Lithuania is excluded as it only provides information on foreign groups with a group size below three. Further, new states have been created in the Eastern European transformation process. In the case of the Czech Republic and Slovakia, I am unable to distinguish between ‘actual’ foreign born and those who report being born in another country without having migrated. Therefore, I also exclude the foreign born living in the Czech Republic who report being born in Slovakia and vice versa.

The final sample includes 6,023 European foreign-born respondents living in private households in 24 European countries (18 Western Europe; 6 Eastern Europe) for whom information on all variables is available. The large majority of respondents (N = 5,884; 97.7%) reside in a Western European country–of whom 81% were born in another Western European country and 19% originate from an Eastern European country covered by the survey. The share of foreign born currently residing in an Eastern European country is small (N = 139; 2.3%) with an equal amount of respondents being born in another Eastern or Western European country. It is noteworthy that a large majority of foreign born (85.1%) reside in an economically more prosperous country compared to their country of origin. However, there is no clear indication that respondents have chosen their country of residence on the grounds of better healthcare standards. In total, 223 groups can be identified with foreign born respondents sharing the same country of origin and country of residence. S1 Table provides further details on the number of respondents for countries and survey rounds used in the analysis. S2 Table gives information on the number of respondents grouped by the country of origin and country of residence; this also offers valuable information on the size of foreign-born groups within the destination country.

### Measures

#### Dependent variable

The evaluation of healthcare services is the main dependent variable. Respondents are asked what they think overall about the state of healthcare services in their country of residence on an 11-point scale, ranging from 0, extremely bad, to 10, extremely good.

#### Independent variables: Country level

I distinguish between the following institutional characteristics of the healthcare system’s ‘production process’ [11]:

‘Monetary input’ is measured by the following indicators available in the OECD database [43]. The total amount of healthcare expenditure (THE) (per capita, constant prices, PPP) reflects the total financial investment in healthcare. The amount of public health expenditure (PHE) as a percentage of THE captures the relative amount of financial resources spent on healthcare by the state or other state-related (non-profit) insurance agencies. For a detailed understanding of the sources of public investments, I further distinguish between the amount of PHE from government schemes (PHE, government) and compulsory contributory health insurance schemes (PHE, insurance), both measured as a percentage of THE. Finally, the amount of out-of-pocket expenditure (OOP) as a percentage of THE provides direct information on the relative financial burden on the healthcare user.

Information on the ‘real input’ dimension, i.e. the supply of healthcare personnel and infrastructure, also comes from the OECD database. I choose three indicators: the number of general practitioners (density per 1000 population) reflects the supply of healthcare personnel for primary (outpatient) healthcare services; the number of specialist medical practitioners (density per 1000 population) yields information on the supply of healthcare personnel for secondary (inpatient and outpatient) care; the number of hospital beds (per 1000 population) indicates the supply of inpatient services [9,20].

I measure institutional regulation of patients’ access to medical care by legal restrictions on (i) the choice of the level of care and (ii) the choice of provider [22]. For this, I use the MISSOC database for 2008 and the WHO Health in Transition country-specific reports, as well as previous research on access regulations and coding schemes [20,22]. To measure the patient’s freedom to choose to consult a GP or to present a health problem directly to a specialist, I distinguish three types of regulation: (i) gatekeeping systems require referral by the GP to guarantee access to specialist care (‘restricted choice’); (ii) skip and pay systems allow direct access to specialist care for an additional charge (‘partly restricted choice’); (iii) free access systems do not require a GP’s referral and do not charge additional fees for the direct consultation of a specialist (‘free choice’). Further, and to assess the patient’s freedom to choose primary healthcare providers, I distinguish between (i) countries that limit the choice of primary care providers to the local area or to providers contracted by the health insurance within the area, and (ii) countries without such restrictions. It is worth noting that some health systems may provide different pathways to accessing health services, and these can vary with the person’s insurance status. For example, persons residing in Switzerland can chose alternative health insurance plans with lower insurance premiums at the expense of restricted access to healthcare services. For the ease of classification, the traditional and statutorily regulated pathway to accessing healthcare services within the public health system is used in this study.

The general attitude towards healthcare services within the native (majority) population is based on ESS data. I calculate the averages of the healthcare evaluations using the dependent variable described above.

S3 Table gives information on all contextual variables at the country level. With the exception of access regulations, all variables report mean values for the time period between 2002 and 2014. Therefore, changes in healthcare settings over time are not considered in the analysis.

#### Independent variables: Group level

The ‘relative’ performance of healthcare systems compared to the country of birth is measured by the differences in institutional characteristics between the country of residence and the country of birth (for all metric variables stated above). I distinguish three categories of access regulation measures: (i) no change in access regulations, (ii) more freedom of choice, and (iii) less freedom of choice in host country than in country of birth. All values at the group level are specific to groups of foreign born in the host country grouped by the country of birth.

This identification strategy has its weaknesses as values reflect the average difference in healthcare performance over a fixed time period (2002 to 2014). Measures may not accurately reflect the performance of healthcare services in the country of origin experienced at the time the foreign born has resided in the country. Consequently, effects are likely to be underestimated, specifically for those foreign born who had left their country of birth before 2002 and have no knowledge of the current state of healthcare in the country of origin. As no information is available on the time and length the respondent has lived in the country of origin, there is no practical solution to this empirical problem.

#### Independent variables: Individual level

To avoid biased estimates due to compositional effects of the study population, I control for the demographic and socio-economic characteristics of the respondent. The respondent’s sex and age function as standard control variables. To control for health needs, I include the respondent’s self-reported health status measured on a 5-point scale, ranging from very bad to bad, fair, good and very good. I introduce socio-economic characteristics, including status of employment (paid work, unemployed, retired, other status) and level of education: completed lower secondary education or less (ISCED1/2, ‘low educated’), upper secondary education and post-secondary non-tertiary education (ISCED 3/4, ‘medium educated’), and tertiary education (ISCED 5, ‘high educated’). I use a subjective income indicator as a proxy for the financial resources available to the household. In the survey, respondents are asked how they feel about their household income today and whether they live comfortably on their present income, cope on their present income, find it difficult on their present income, or find it very difficult on their present income.

I also control for characteristics specific to the study population likely to affect healthcare evaluations. To test hypothesis 3a and 3b, I include a dummy variable of the length of stay in the country of residence to estimate whether those who recently entered the country (less than six years ago) evaluate healthcare services differently from those in the country longer (more than five years). Further, I control for experiences of discrimination because of the respondent’s colour/race, nationality, language or religion. Negative experiences of the foreign born may affect their general perception of the host country’s society and its public institutions. These experiences can cause bias in the estimates of institutional effects, if they are not controlled for in the analysis. S4 Table provides the mean values/proportions of all independent variables at the individual level.

### Data analysis

I apply multilevel modelling techniques to estimate the effects of individual-, group-, and country-level characteristics and their interaction. Unlike conventional regression analysis, multilevel models account for a hierarchical or nested data structure, whereby observations at lower levels are nested in higher order units. I distinguish three analytical levels: the ‘macro level’ (country of residence; N = 24), the ‘meso level’ (groups of foreign born in country of residence by country of birth, N = 223), and the ‘individual level’ (the foreign-born respondent, N = 6,023). With an intraclass correlation of .17 (design effect: 44.24) at the country level and .03 (design effect: 1.81) at the group level, the use of multilevel models for the analysis is highly recommended.

Random intercept models allow intercepts to vary across countries and groups. Variations in intercepts can be explained (i) by country level predictor variables, i.e. the institutional characteristics of health services in the country of residence (to test hypotheses 1a and 1b), (ii) by group level predictor variables, i.e. differences in institutional characteristics between the country of residence and the country of birth (to test hypotheses 2a and 2b), and (iii) by individual level variables, i.e. the length of stay in the country of residence (to test hypothesis 3a), as well as demographic and socio-economic characteristics of the individual to control for the compositional variation of the foreign-born population [44].

Random slope or random coefficients models allow slopes to vary across higher-level units. Variations in slopes can be explained by higher-level predictor variables modelled as cross-level interactions between a higher-level variable and individual attributes whose effects are allowed to vary between higher level units. I use a random coefficient model to measure cross-level interactions between group-level predictor variables and the length of time a respondent has lived in the country (1 = ‘< 6 years’; 0 = ‘> 5 years’) and to test hypothesis 3b. The variation in healthcare evaluations between recent arrivals and those living in the country for a longer period is stronger and significant at the group level (Variance = .18, SE = .10); it is weaker and non-significant at the country level (Variance = .09, SE = .09).

I perform step-wise multilevel regression analyses to empirically test the hypotheses (as illustrated in Table 1). At all stages, I control for the respondent’s characteristics at the individual level and include a time variable to control for time specific trends. Please note that I ran the analyses using time dummies to control for each survey round/wave first. I discovered a positive and continuous time trend. To reduce the number of parameters, I included a continuous time-trend variable for the analyses presented here. When estimating institution-specific effects (Table 2), I also control for the direct and interactional effects of health expenditure at the country and group level (as presented in Table 1). This ensures that effects of other institutional characteristics are not spurious and not the result of the financial resources flowing into the system. Please note that total health expenditure is closely related to the economic development of a country; thus, it captures other aspects of a country’s economic and social welfare as well, which are not additionally controlled for in this analysis. I estimate all models using maximum likelihood with robust standard errors using Mplus, version 8 [45]. Results are presented for each institutional indicator separately; consequently, the description of results does not follow the same sequential order as the hypotheses presented above.

**Table 1:** Effect of total health expenditure on healthcare ratings.

	Model 0		Model 1		Model 2	
	β	SE	β	SE	β	SE
Intercept	6.02***	.27	6.05***	.24	6.04***	.23
*Country Level*						
Total health expenditure (per capita, constant, PPP)			.45***	.12	.41**	.12
*Group Level*						
Difference in total health expenditure (country of residence vs origin)			.16**	.05	.14*	.05
Difference in total health expenditure x recent migration					.03	.08
*Individual Level*						
Recent migration (0 = > 5 years in country)	.41**	.15	.41**	.15	.46**	.15
*Control Variables*: *Individual level*						
Experienced discrimination (0 = no)	-.21*	.10	-.22*	.10	-.22*	.10
Female (0 = male)	-.28**	.09	-.28**	.09	-.29**	.08
Age	.01***	.00	.01***	.00	.01***	.00
Subj. health (metric, 0 = fair)	.22***	.05	.23***	.05	.23***	.05
Education: medium (0 = low)	-.30***	.06	-.30***	.06	-.30***	.07
Education: high	-.42***	.07	-.41***	.07	-.41***	.07
Subj. income: coping (0 = living comfortably)	-.25***	.05	-.26***	.05	-.27***	.05
Subj. income: (very) difficult	-.47***	.13	-.49***	.13	-.49***	.13
Employment: unemployed (0 = employed)	.35*	.15	.36*	.15	.36*	.15
Employment: not in labour force	.31***	.06	.31***	.06	.31***	.07
Time trend (2002–2014)	.13***	.02	.12***	.02	.13***	.02
*Variance Components*						
Country Level: variance	1.10***	.29	.57**	.18	.64**	.19
Group Level: variance	.19***	.04	.15***	.03	.12***	.03
Individual Level: variance	4.88***	.30	4.89***	.30	4.85***	.31
Country Level: variance (recent migration)					.11	.09
Country Level: covariance (intercept/recent migration)					-.21*	.09
Group Level: variance (recent migration)					.15	.11
Group Level: covariance (intercept/recent migration)					.08	.06
AIC	26853		26832		26822	
BIC	26960		26953		26976	

European Social Survey, round 1–7, sample population: foreign born respondents in Europe; multilevel analysis based on three levels: individuals/foreign born (N = 6023), groups of foreign born (N = 223), countries of residence (N = 24); table reports unstandardized coefficients (β) and standard errors (SE); coefficient of recent migration was fixed in Model 0 and Model 1 and randomized in Model 2

* *p* < 0.05

** *p* < 0.01

*** *p* < 0.001 (two-tailed test)

**Table 2:** Additional effects of other institutional healthcare characteristics on healthcare ratings.

Institutional Healthcare Characteristics	Level of Effect		Model 1			Model 2	
		Β	SE	Var.	β	SE	Var.
A Effects of Monetary Input							
Public Health Expenditure (% THE)	Country level	.03	.03	.54	.04	.02	.62
	Group level	.01	.01	.14	.01	.01	.12
	Cross-level interaction with recent migration				.01	.01	.16
Public Health Expenditure of Government Schemes (% THE)	Country level	.00	.01	.53	.00	.01	.62
	Group level	-.01**	.00	.12	-.01*	.00	.10
	Cross-level interaction with recent migration				-.00**	.00	.11
Public Health Expenditure of Compulsory Insurance Schemes (% THE)	Country level	.00	.01	.50	-.00	.01	.58
	Group level	.01**	.00	.11	.01**	.00	.10
	Cross-level interaction with recent migration				.01**	.00	.12
Out-of-Pocket Expenditure (% THE)	Country level	-.01	.02	.56	-.01	.02	.62
	Group level	-.02^+^	.01	.13	-.02+	.01	.11
	Cross-level interaction with recent migration				-.01	.01	.15
B Effects of Real Input							
Density of GPs (per 1000)	Country level	1.21^+^	.71	.40	1.25*	.58	.46
	Group level	.35**	.12	.12	.24^+^	.15	.11
	Cross-level interaction with recent migration				.32	.22	.09
Density of Specialists (per 1000)	Country level	.01	.46	.57	-.02	.47	.64
	Group level	.02	.08	.15	.02	.07	.12
	Cross-level interaction with recent migration				.07	.19	.16
Density of Hospital Beds (per 1000)	Country level	.02	.12	.52	-.05	.10	.61
	Group level	.10**	.04	.11	.09*	.04	.09
	Cross-level interaction with recent migration				.12***	.03	.10
C Access Regulation							
Restricted Access to Specialist Care Services	Country level: referral by GP (0 = no restriction)	.02	.50	.55	.13	.46	.61
	Country level: skip & pay	-.28	.63		-.13	.61	
	Group level: more freedom (0 = no change)	.24^+^	.14	.14	.30**	.11	.11
	Group level: less freedom	-.28**	.09		-.13	.11	
	Interaction: more freedom x recent migration				-.27	.26	.13
	Interaction: less freedom x recent migration				-.71**	.22	
Provider Restriction for Primary Care Services	Country level: provider restriction (0 = no restriction)	.76**	.29	.56	.81**	.28	.62
	Group level: more freedom (0 = no change)	.30*	.12	.11	.23^+^	.12	.10
	Group level: less freedom	-.76***	.09		-.76***	.12	
	Interaction: more freedom x recent migration				.57***	.12	.07
	Interaction: less freedom x recent migration				-.05	.26	
D Soft Institutions							
Native’s Opinion on Healthcare (mean values)	Country level	.67***	.13	.10	.67***	.14	.14
	Group level	.13^+^	.07	.14	.14*	.07	.11
	Cross-level interaction with recent migration				-.14	.09	.15

European Social Survey, round 1–7, sample population: foreign born respondents in Europe; multilevel analysis based on three levels: individuals/foreign born (N = 6023), groups of foreign born (N = 223), countries of residence (N = 24); table reports unstandardized coefficients (β), standard errors (SE) and residual variances (Var.) at the country and group level and for the random slope coefficient (recent migration); analysis based on Table 1; all analyses control for experienced discrimination, length of stay in country of residence, demographic (sex, age) and socio-economic characteristics (education, income, employment status) and year of survey at the individual level, and the direct and interactional effects of the absolute and relative amount of total health expenditure (per capita, constant prices, PPP) at the country and group level (see Table 1)

^+^ *p* < 0.10

* *p* < 0.05

** *p* < 0.01

*** *p* < 0.001 (two-tailed test)

Note that to check robustness, I conducted additional analyses using different sample sizes. Scholars point out that the statistical power of multilevel analysis increases with the number and size of clusters [46–48]. Results of this study are based on a minimum cluster size of three at the group level and a total of 223 clusters. I re-ran the analyses with a cluster size fixed to a minimum of one, five, or seven. This increased (cluster size: minimum of one) or reduced (cluster size: minimum of five or seven) the number of clusters at the group level accordingly. Results are presented in S5 Table. If not stated otherwise, findings are robust and not sensitive to the size or number of clusters at the group level.

## Results

Table 1 shows the results of the step-wise multi-level regression analysis for the effects of total health expenditure on healthcare evaluations of the foreign born. The baseline model (Model 0) includes all individual level characteristics and specifies the amount of unexplained variance of healthcare evaluations after controlling for the sample composition. Hypothesis 1a, i.e. the direct institutional effect of total health expenditure in the country of residence, and hypothesis 2a, i.e. the direct referential effect of the difference in total health expenditure between the country of residence and the country of origin, are tested in Model 1 using a multi-level random intercept model. To test hypothesis 3b, a cross-level interaction effect is included in the analysis (see Model 2) on the difference in total health expenditure at the group level and the length of time a respondent has lived in the country using multi-level random coefficient analysis.

In line with hypothesis 1a, the results show that healthcare evaluations of the foreign born are increasingly positive with higher amounts of THE (per capita, constant prizes, PPP) in the country of residence (Table 1, Model 1). The effect is robust and explains 36% of the variance in healthcare evaluations at the country level. In support of hypothesis 2a, healthcare evaluations also depend on the amount of health expenditure relative to the country of origin. The results show that the higher the financial expenditure in the country of residence compared to the country of origin, the higher the healthcare rating. These relative financial resources explain 29% of the variance at the group level (Table 1, Model 1). Further, and consistent with hypothesis 3a, the foreign born who recently entered the country are more positive about healthcare services than those in the country for a longer period (Table 1, Model 0–2). However, contrary to hypothesis 3b, comparisons of financial resources with the country of origin have no significantly stronger impact on the assessments of recent arrivals than on those of earlier arrivals (Table 1, Model 2).

Do other institutional characteristics of the healthcare system add to the explanation of healthcare evaluations of the foreign born? Results on the total health expenditure form the groundwork for the subsequent analyses of the specific additional impact of the various sources of health expenditure (‘monetary input’), supply of healthcare personnel and infrastructure (‘real input’), institutional regulations of patients’ access to medical care (‘institutional set-up’), and the opinions of the native born on healthcare (‘soft institutions’) on healthcare ratings of the foreign born. The analysis follows the same step-wise procedure as indicated in Table 1; additional institutional indicators are included one-by-one in the analysis. In total, Table 2 reports the results of 20 regression models, testing the direct effects (Model 1) and interactional effects (Model 2) of 10 additional institutional characteristics. The models control for individual level characteristics, as well as the direct and interactional effects of the total amount of health expenditure. Please note that for reasons of space, only the direct effects of the additional institutional indicators at the country and group level (testing hypotheses 1a/b and 2a/b), as well as the interactional effects between the additional institutional indicator at the group level and the length of residency at the individual level (testing hypothesis 3b), are presented in Table 2.

### Sources of monetary input

Contrary to hypothesis 1a, results show no indication of a robust and significant effect of the amount of public health expenditure (PHE–total/government schemes/insurance schemes, % of THE) or out-of-pocket expenditure (% of THE) on healthcare evaluations (Table 2, Section A, Model 1). Whether the foreign born experience changes in out-of-pocket expenditure or public financing schemes is more important for their healthcare ratings. In support of hypothesis 2a, the foreign born who now spend more of their own money on healthcare services rate these services less positively than those who do not. Interestingly, the foreign born also rate systems financed by compulsory contributory health insurance schemes higher if the system in their country of origin is government (tax) financed and vice versa. Together with differences in THE, these relative effects explain up to 57% of the variations in healthcare ratings between the foreign born at the group level. Changes in financing schemes have a significantly stronger effect if the foreign born have recently entered the country of residence (Table 2, Section A, Model 2), consistent with hypothesis 3b. Whether this is also the case for out-of-pocket expenditure remains unclear, as results vary with the size and number of clusters at the group level (S5 Table).

### Real input

In line with hypothesis 1a, results suggest that the foreign born are more positive about healthcare services when primary care services are more dense (Table 2, Section B, Model 1). Together with the amount of THE, the effect of primary healthcare services explains 56% of the variance at the country level. Contrary to hypothesis 1a, but in line with previous research, no significant effects are found for the provision of specialist and inpatient care in the host country. Further, and in support of hypothesis 2a, the foreign born rate healthcare systems of the country of residence more positively if they have a greater density and supply of primary and inpatient services than they did in the country of origin; this explains up to 38% of the variance at the group level. Consistent with hypothesis 3b, the effect of the relative provision of hospital beds is significantly stronger if the foreign born have entered the country recently, i.e. within the past five years (Table 2, Section B, Model 2). Whether this is also the case for the provision of primary care services remains unclear, as results vary with the size and number of clusters at the group level (S5 Table in supplementary material). The absolute and relative density of specialist care is not significantly related to healthcare ratings.

### Access regulations

Although the freedom of choice in accessing specialist services does not explain differences in healthcare evaluations between countries, having the freedom to choose a basic provider does matter (Table 2, Section C, Model 1). In fact, contrary to hypothesis 1a, the foreign born rate healthcare services more positively if they live in a country with provider restrictions, but effects are not robust and vary with the size and number of clusters at the group level. Yet knowledge and experience of alternative regulatory systems matter. The foreign born who experience a change in regulations show the predicted effects in support of hypothesis 2a: the more choice they are given when selecting a provider relative to their country of origin, the higher their healthcare ratings; conversely, a loss of choice leads to lower healthcare ratings. Results on access to specialist care point in the same direction, suggesting the less freedom the foreign born have when accessing specialist care, the lower their healthcare ratings; more freedom is associated with higher healthcare ratings. These results vary slightly with the size and number of clusters at the group level and are more robust if the length of stay in the destination country is considered in the analysis as well. In support of hypothesis 3b, the foreign born who entered the country of residence recently rate healthcare ratings more positively, if they are given more freedom to choose a provider than those in the country for longer. They are also more negative in their healthcare ratings if they have less freedom in accessing specialist care. In fact, this loss in freedom seems to affect healthcare ratings of recent arrivals only, as a significant effect is no longer observable for those longer in the country (Table 2, Section C, Model 2).

### Opinions of the native born on healthcare

Without a doubt, and in support of hypotheses 1b and 2b, the foreign born are more positive about the healthcare services in the country of residence when these are more positively perceived by the native population and when perceptions are more positive than those of the native population in the country of origin. Together with the amount of THE, these factors explain up to 87% percent of the country level variations in the attitudes of the foreign born and 35% of the variations at the group level (Table 2, Section D, Model 1). Contrary to hypothesis 3b, the effects do not show a stronger impact on the assessments of recent arrivals (Table 2, Section D, Model 2).

## Discussion

Debates on healthcare policy in Europe often cite public opinion [7,8,10]. Building on this line of research, this study explored healthcare ratings from the perspective of the foreign born, i.e. those residing in a European country in which they were not born. By studying the foreign born it addresses the problem of endogeneity in public opinion research and provides an alternative approach to explore the impact of socialisation and adaptation processes, especially experience and knowledge of alternative institutional arrangements, on public opinion. The study considered the impact of healthcare institutions on healthcare ratings using data from the European Social Survey 2002–2014. The final sample comprised 6,023 foreign-born respondents living in private households in 24 European countries.

### Institutional characteristics of the country of residence

Findings partly support hypothesis 1a which expected the foreign born to evaluate healthcare services in the destination country according to the degree to which institutions meet their needs and interests. Overall, results indicate some institutional characteristics of the country of residence determine how the foreign born rate healthcare services. In line with research on native populations [11,12,20,21], this study finds a strong and positive association between healthcare evaluations, the amount of total healthcare expenditure and the density of general practitioners. Also in line with previous research on native populations [11,20], there is no significant association between other institutional characteristics and healthcare ratings, with one exception: contrary to the initial hypothesis (H1a), the foreign born evaluate healthcare services more positively if they live in a country which limits the choice of primary care providers to the local area or to providers contracted by the health insurance within the area. Although this finding is not robust and varies with the number and size of clusters considered at the group level, it deserves more attention. Restrictions in provider choice are observed in countries like Austria, Denmark, Finland, the UK, and the Netherlands–countries that often score high on other healthcare characteristics–as well as Italy and Portugal. Similar results are reported by Popic and Schneider for older birth cohorts (born before 1951) [20]. They found healthcare ratings to be higher in countries where registration with a GP was obligatory. The authors reasoned that respondents–specifically older birth cohorts–valued the positive side effects of such regulations (e.g. the stability and continuity of the doctor-patient relationship), rather than perceiving such regulations as a limitation of their patient rights.

Further, this study’s research design allows an examination of the association between ‘soft’ institutional indicators, i.e., how the native born evaluate healthcare services, and the opinions of the foreign born. In support of hypothesis 1b, healthcare evaluations of the foreign born are closely associated with the average ratings of native-born persons in the host country. This finding is consistent with the expectation that the degree to which the majority population appreciates healthcare services may shape the opinions of the foreign born.

### Institutional characteristics of the country of origin

Social comparisons are a fundamental psychological process; they help individuals to evaluate themselves and the world around them [49]. In line with findings from migration research [32,34,42,50], and in support of hypotheses 2a and 2b, results show that referential standards and comparisons with the country of origin are particularly relevant in the healthcare ratings of the foreign born. With only a few exceptions (public health expenditure, density of specialists), relative performance indicators show the predicted effects on healthcare evaluations. Most interestingly, the analyses highlight the significance of prior experiences of access regulations and public financing schemes. While research on native populations does not find an empirical association between healthcare evaluations and institutions that regulate access to medical care [11,20], this study’s findings suggest that the foreign born who have previously been more restricted in accessing specialist care or selecting a primary care provider acknowledge their new freedom when rating healthcare services in the destination country; those who experience the new system as more restrictive than their home country evaluate healthcare services less positively. Further, results show experiences with different public financing schemes matter. The foreign born rate systems primarily financed by compulsory contributory health insurance schemes higher if they have previously experienced government (tax) financed national healthcare systems and vice versa. This finding supports prior research claiming that individuals living in countries with multi-payer, contribution-based Social Health Insurance (SHI) systems are more satisfied with their healthcare services than individuals living in countries with single-payer, tax-financed National Healthcare Service (NHS) systems [11]. In sum, findings show: the better the relative performance of healthcare services in the country of residence compared to the country of origin, the more positive the evaluation of healthcare services.

### Adaptation effects and length of residency

In support of hypothesis 3a, and in line with previous research on migrants’ opinions of public institutions [34,40,41], the foreign born who recently entered the country are more positive in their healthcare ratings than those who have lived there longer. Research often suggests that an initial optimism and excitement makes immigrants more positive of the host country’s public institutions in the first few years after their arrival. Over time, they adapt to the rules and regulations of the host society and adopt attitudes similar to the majority population [42]. Although the influence of referential structures is often observable for all foreign-born groups independent of the length of time they have lived in the destination country, there is some minor support for hypothesis 3b. Changes in public financing schemes, the provision of inpatient services, and some access regulations (i.e. less freedom in accessing specialist care and more freedom in choosing a primary healthcare provider) have a stronger effect on recent arrivals than on those who have lived in the country longer (more than five years). A loss in freedom accessing specialist care seems to affect healthcare ratings of recent arrivals only.

### Implications for health policy research

We can say with some confidence that the amount of financial resources and the density of primary healthcare services are associated with the public’s opinion of healthcare, i.e. how people think and feel about the healthcare system in the country where they live [11,12,20,21]. Other characteristics seem to be of less relevance for healthcare ratings if individuals do not know better and have no knowledge or experience of alternative healthcare arrangements. The foreign born who have lived in different healthcare settings are aware that healthcare services can be arranged differently and depend on political will. This knowledge shapes their evaluations. This study’s findings suggest the foreign born evaluate systems more positively if these systems are sufficiently well financed and place less financial burden on the public compared to systems in the country of origin and if they are primarily funded by compulsory contributory health insurance schemes. Further, when there is a higher density of primary care and inpatient services in the host country than in the country of origin, and when regulations provide access to medical care freely and without restrictions as to provider, the approval rates go up. Also important but less acknowledged are ‘soft’ institutional factors, such as the reputation healthcare services enjoy within the larger society. These factors may have secondary effects on the foreign born and seem to generate support or disapproval of health policy decisions.

### Implications for public opinion research

The empirical results have implications for public opinion research as well. Neo-institutionalist theory tells us that institutions endow meaning and shape preferences and expectations that produce public support of the status quo [24]. Others emphasize the importance of knowledge and experience, as well as social comparison processes and referential standards, to the evaluation process [51–53]; some suggest evaluations are a direct function of the discrepancy between expectations and perceptions of the ‘status quo’ [54,55]. This study addresses the problem of endogeneity, namely that public institutions shape preferences and produce a somewhat one-dimensional or ‘naïve’ perspective of the status quo, by examining the opinions of the foreign born. This way, biases caused by institutional feedback effects can be minimized and adaptation as well as socialisation processes of the foreign born, who have lived in different institutional settings, can be studied simultaneously. Findings support the expectation that the foreign born make use of their experience and knowledge of alternative healthcare systems to evaluate the current state of affairs. This has implications for public opinion: specifically, it suggests that increased knowledge (and actual experience) will change views on public institutions, especially with rising mobility across European countries. Furthermore, findings show that only few institutional characteristics in the destination country affect performance evaluations of the foreign born. As results are fairly similar to those found for the general population, it suggests that institutional feedback effects do not seem to play an essential role for evaluative attitudes, i.e. performance evaluations of public institutions such as healthcare. Last but not least, findings indicate that attitudes are not (always) stable cognitive constructs but may vary over an individual’s life course (e.g. with the length of time the foreign born has resided in the destination country [41]).

### Limitations

This study is not without limitations. For one thing, the findings may suffer from selection effects. The ESS does not apply a specific sampling scheme for the foreign born. Since questionnaires are prepared in the language(s) used by at least five per cent of the population in any given country, a sufficient level of language proficiency by respondents in minority groups is required to answer the questionnaire. Further, the ESS provides data on a limited number of European countries only. The majority of the study population was born in one of the 18 Western European countries covered by the survey and continued to reside in a Western European country; data on Eastern Europe is provided for 6 countries only and falls short on providing sufficient information on countries that report a high number of emigrated citizens (such as Romania, Albania, Croatia, Bosnia and Herzegovina). Thus, findings are clearly not based on a representative sample of the foreign born residing in Europe, and certain groups of foreign born are more likely to be excluded from the survey than others. This reduces the generalizability of results and restricts them to the countries and groups of foreign born investigated in this study.

In addition, because of the small number of foreign-born respondents, I use the pooled sample of all seven survey rounds of the ESS for the analysis. Although I control for a common time trend in healthcare evaluations across European countries, I cannot explore healthcare evaluations from a longitudinal perspective or study the consequences of institutional change on them. More research is certainly warranted, especially given the ongoing reform processes in the field of healthcare [56], not to mention changes in healthcare financing and provision following the recent economic crisis [57]. At the same time, healthcare evaluations of the foreign born may be less stable over time than those observed for the native born. As of now, no straightforward answer can be given on how healthcare evaluations will differ if the time perspective is considered in the analyses.

At the same time, I am unable to include information on institutional systems other than those in the country of residence and the country of birth. No information is available on whether the respondent has lived in other countries and institutional settings between the time of emigration from the country of origin and the time of arrival in the destination country. I have most likely underestimated the effect of the country of origin as well. The foreign born who migrated before 2002 may have experienced different conditions in the country of birth than those captured in the analysis. Findings are significant for most relative performance indicators, but effect sizes might have been larger if measures had considered the performance of healthcare at the time of actual experience. Advances in technological change and the implementation of quality standards in healthcare monitoring have improved the availability and effectiveness of healthcare services in most European countries over the past decades. However, cost containment strategies that foresaw cuts in public budgets and personnel as well as privatisation of healthcare services may have been experienced already by the foreign born at the time of emigration. To predict the bias in the observed results for those who left their country of origin a long time ago and who are unaware of the current state of healthcare in the country of birth, more specific data on the individual and his/her migration history is needed.

Further, I cannot control for a respondent’s direct experience of healthcare services and the type of insurance status in the country of residence. Studies report immigrants to be healthier but also more vulnerable to certain diseases [58]. They often face specific barriers in accessing healthcare services [59,60] and lack information about entitlements and the organisation of healthcare services in the destination country [61,62]. Although I control for individual level characteristics, such as demographic and socio-economic background variables, health needs, and experiences of discrimination in the country of residence, more research is needed on how these specific experiences shape the opinions of the foreign born on healthcare services.

Last but not least, this study’s research design may raise concerns of endogeneity, if the foreign born have entered the country of residence for reasons of healthcare. Indeed, the ‘welfare magnet hypothesis’ suggests immigrants choose their destination according to the generosity of welfare benefits [63], but research is still divided on its empirical applicability. Unfortunately, I do not have information on what caused the foreign born in the sample to migrate, nor do I have information on their reasons for selecting the country of residence. The study sample includes persons originally from Europe now residing in a European country they were not born in. Whilst healthcare might be one of the motives for migrants from developing countries to move to a European country, I expect the study population to be less likely to have left their country of origin to receive healthcare in the destination country. This is supported by healthcare indicators measuring the differences in healthcare between the country of residence and the country of origin. No indications are found for respondents having chosen their country of residence on the grounds of better healthcare standards, as there is no severe skewness in the distribution of these indicators observed. However, more research on the motives for migration is surely warranted when studying opinions of the foreign born.

## Conclusion

Despite these limitations, this study provides a deeper understanding of the institutional determinants of healthcare evaluations. By studying healthcare ratings from the perspective of the foreign born, i.e. those who have not been socialized in the system they are asked to evaluate and who are likely to apply different frames of reference, this study shows that socialisation processes within institutional settings matter to the formation of attitudes. The support of public institutions, in this case healthcare, is not just a matter of personal resources and interests. It also depends on the institutional structures, especially the experience and knowledge of alternatives. The knowledge that institutions can be arranged differently shapes a person’s judgement of them.

## Supporting information

S1 Table Number of observations by country of residence and survey round. (PDF)

S2 Table Number of observations by country of residence and country of birth. (PDF)

S3 Table Institutional characteristics of healthcare systems, mean values, 2002–2014. (PDF)

S4 Table Individual-level characteristics, mean values/proportions. (PDF)

S5 Table Effects of institutional healthcare characteristics on healthcare ratings–with varying cluster sizes at group level. (PDF)
