# Supplementary material for: Beyond endogeneity in analyses of public opinion: Evaluations of healthcare by the foreign born across 24 European countries
Source: PLoS One. 2020 Jun 1;15(6):e0233835. doi: 10.1371/journal.pone.0233835 (PMC7263607; doi:10.1371/journal.pone.0233835)
Supplement: S1 Table — (PDF) [file pone.0233835.s001.pdf]

S1 Table: Number of observations by country of residence and survey round

Country	2002	2004	2006	2008	2010	2012	2014	Total
AT	63	67	65	-	-	-	45	240
BE	76	70	66	80	46	55	54	447
CH	208	211	196	233	137	149	163	1,297
CZ	6	9	-	2	3	2	3	25
DE	55	63	61	49	45	50	77	400
DK	10	20	25	34	25	10	29	153
EE	-	5	9	5	-	1	3	23
ES	18	18	18	36	23	20	8	141
FI	15	8	9	11	9	14	21	87
FR	-	-	48	51	18	23	30	170
GB	42	30	46	56	51	49	41	315
GR	13	26	-	12	10	-	-	61
HU	2	3	2	2	1	1	-	11
IE	96	80	116	155	167	154	131	899
IS	-	6	-	-	-	5	-	11
IT	5	6	-	-	-	3	-	14
LU	257	304	-	-	-	-	-	561
NL	45	31	31	35	29	23	28	222
NO	39	26	51	54	47	45	44	306
PL	8	10	1	6	1	-	-	26
PT	6	7	2	5	5	5	3	33
SE	90	84	93	106	45	50	59	527
SI	8	7	7	7	2	0	-	31
SK	-	4	9	8	-	2	-	23
Total	1,062	1,095	855	947	664	661	739	6,023

Note: European Social Survey, rounds 1–7, sample population: foreign born respondents in Europe; table reports number of respondents by country of residence and year of survey. For further information on sample selection see methods section of the manuscript.
