# Supplementary material for: Beyond endogeneity in analyses of public opinion: Evaluations of healthcare by the foreign born across 24 European countries
Source: PLoS One. 2020 Jun 1;15(6):e0233835. doi: 10.1371/journal.pone.0233835 (PMC7263607; doi:10.1371/journal.pone.0233835)
Supplement: S2 Table — (PDF) [file pone.0233835.s002.pdf]

S2 Table: Number of observations by country of residence and country of birth

Country of Origin:	AT	BE	CH	CZ	DE	DK	EE	ES	FI	FR	GB	GR	HU	IE	IS	IT	LT	LU	NL	NO	PL	PT	SE	SI	SK	Total
Country of Residence:																										
AT	-	-	4	17	110	-	-	-	-	6	5	3	21	-	-	24	-	-	4	-	28	-	-	9	9	240
BE	-	-	3	3	32	-	18	-	-	142	7	7	3	-	-	85	-	5	98	-	21	20	3	-	-	447
CH	104	16	-	17	446	6	45	-	8	152	42	9	19	5	-	226	-	-	35	-	25	112	14	6	10	1,297
CZ	-	-	-	-	-	-	-	-	-	-	-	-	9	-	-	-	-	-	-	-	16	-	-	-	-	25
DE	26	-	6	34	-	4	16	-	-	12	15	10	10	3	-	34	4	-	20	-	189	6	-	7	4	400
DK	-	4	-	-	36	-	4	-	6	8	14	-	4	4	11	3	-	-	7	14	19	-	19	-	-	153
EE	-	-	-	-	6	-	-	-	6	-	-	-	-	-	-	-	11	-	-	-	-	-	-	-	-	23
ES	-	5	4	-	13	3	-	-	4	32	27	-	-	-	-	14	4	-	7	-	8	17	-	-	3	141
FI	-	-	-	-	7	-	4	42	-	-	6	-	-	-	-	-	-	-	4	-	3	0	21	-	-	87
FR	4	15	6	-	18	-	26	-	-	-	16	4	-	-	-	24	-	-	-	-	10	47	0	-	-	170
GB	-	5	-	5	45	-	11	3	3	22	-	-	3	96	-	22	5	-	8	-	71	8	5	-	3	315
GR	3	4	-	3	35	-	-	-	-	-	9	-	-	-	-	3	-	-	-	-	4	-	-	-	-	61
HU	-	-	-	-	-	-	-	-	-	-	-	-	-	-	-	-	-	-	-	-	-	-	-	-	11	11
IE	-	-	-	10	26	4	13	8	-	29	498	-	11	-	-	13	46	-	10	-	223	3	-	-	5	899
IS	-	-	-	-	4	7	-	-	-	-	-	-	-	-	-	-	-	-	-	-	-	-	-	-	-	11
IT	-	-	3	-	-	-	-	-	-	8	-	-	-	-	-	-	-	-	-	-	3	-	-	-	-	14
LU	3	77	3	-	55	5	5	-	-	109	7	-	3	-	-	68	-	-	30	-	3	193	-	-	-	561
NL	5	41	-	-	72	-	7	-	-	14	30	5	6	4	-	9	-	-	-	-	24	5	-	-	-	222
NO	-	3	-	3	36	48	-	-	7	6	30	-	4	-	7	4	8	-	16	-	61	-	73	-	-	306
PL	-	-	-	-	23	-	-	-	-	3	-	-	-	-	-	-	-	-	-	-	-	-	-	-	-	26
PT	-	4	-	-	7	-	4	-	-	18	-	-	-	-	-	-	-	-	-	-	-	-	-	-	-	33
SE	7	-	6	7	56	45	6	14	214	4	18	8	18	-	7	8	3	-	8	59	39	-	-	-	-	527
SI	6	-	-	-	10	-	-	-	-	4	-	-	-	-	-	11	-	-	-	-	-	-	-	-	-	31
SK	-	-	-	-	-	-	-	-	-	-	-	-	18	-	-	-	-	-	-	-	5	-	-	-	-	23
Total	158	174	35	99	1,037	122	159	67	248	569	724	46	129	112	25	548	81	5	247	73	752	411	135	22	45	6,023

Note: European Social Survey, rounds 1–7, sample population: foreign born respondents in Europe; table reports number of respondents by country of residence and country of birth. Please note that new states have been created in the Eastern European transformation process. In the case of the Czech Republic and Slovakia, I am unable to distinguish between 'actual' foreign born and those who report being born in another country without having migrated. Therefore, I exclude the foreign born living in the Czech Republic who report being born in Slovakia and vice versa. Turkey, Israel and Russia are excluded from the analysis as they are not considered European. Cyprus, Croatia, Bulgaria and Ukraine are excluded as no information on country level characteristics is available in the OECD database. Further, I exclude groups of foreign born with less than three respondents to avoid biases in the estimation process. Lithuania is excluded as it only provides information on foreign groups with a group size below three. Further, I restrict the sample to the foreign born who entered the destination country at the age of 18 and above. This ensures respondents entered as adults and experienced other healthcare services for a considerable time. Unfortunately, for the first four rounds of survey, the age of immigration to the country of residence must be estimated using categorical information (less than 1 year, 1 to 5 years, 6 to 10 years, 11 to 20 years, and more than 20 years) on the length of time the foreign-born person has resided in the country of residence. For further information on sample selection see methods section of the manuscript.
