# Supplementary material for: Beyond endogeneity in analyses of public opinion: Evaluations of healthcare by the foreign born across 24 European countries
Source: PLoS One. 2020 Jun 1;15(6):e0233835. doi: 10.1371/journal.pone.0233835 (PMC7263607; doi:10.1371/journal.pone.0233835)
Supplement: S3 Table — (PDF) [file pone.0233835.s003.pdf]

S3 Table. Institutional characteristics of healthcare systems, mean values, 2002-2014

	Monetary Input					Real Input			Access Regulation		Evaluation
	THE per cap., cons., PPP	PHE, % THE	PHE, gov. % THE	PHE, ins. % THE	OOP % THE	GP per 1000*	Specialist per 1000	H-beds per 1000	GP geo. restriction	Access to specialist+	Native Evaluation
AT	4,062	75.55	30.59	45.13	18.13	0.77	2.29	7.69	1	0	6.60
BE	3,757	76.35	10.99	65.58	18.59	1.15	1.71	6.77	0	2	7.30
CH	5,428	61.66	19.49	42.17	28.88	0.71	2.33	5.23	0	0	6.62
CZ	1,794	85.28	6.75	78.14	13.22	0.71	2.85	7.18	0	0	5.32
DE	4,146	80.01	7.00	73.01	13.63	0.66	2.08	8.39	0	2	4.91
DK	4,208	83.97	83.97	0.00	14.29	0.70	1.50	3.60	1	1	6.29
EE	1,255	76.18	10.48	65.67	21.69	0.70	2.19	5.46	0	1	5.10
ES	2,638	72.45	67.60	4.97	22.11	0.73	2.29	3.21	0	1	5.59
FI	3,250	74.77	60.39	14.37	19.56	1.13	1.67	6.26	1	1	6.81
FR	3,766	78.58	3.81	74.71	7.37	1.61	1.71	6.86	0	2	6.03
GB	2,904	81.40	81.37	0.02	11.05	0.75	1.78	3.32	1	1	5.66
GR	2,433	62.93	27.96	36.00	32.72	0.19	3.63	4.65	0	0	3.66
HU	1,614	68.54	9.01	59.45	27.00	0.34	1.76	7.42	0	2	3.63
IE	3,895	76.62	69.28	0.29	12.85	0.59	1.14	4.13	0	2	4.00
IS	3,541	81.39	53.24	28.15	17.30	0.58	2.24	3.53	0	0	5.97
IT	3,028	76.81	76.65	0.16	21.94	0.79	2.94	3.78	1	1	4.73
LU	6,011	83.72	8.30	75.41	11.45	0.82	1.91	5.48	0	0	6.80
NL	4,257	77.17	4.63	73.51	9.79	0.71	1.64	4.53	1	1	6.00
NO	5,031	83.99	71.18	12.91	15.72	0.77	1.59	4.58	0	1	5.97
PL	1,193	70.20	7.11	63.07	25.55	0.18	1.82	6.60	0	1	3.62
PT	2,476	69.08	68.12	0.97	25.16	0.50	1.95	3.45	1	1	3.97
SE	3,756	82.49	82.49	0.00	16.41	0.61	2.03	2.82	0	0	5.73
SI	2,215	72.68	3.25	69.37	12.67	0.44	1.86	4.71	0	1	5.05
SK	1,614	76.16	6.55	67.02	21.24	0.42	2.58	6.55	0	1	4.16

Note: *Monetary and real input*: based on OECD database (http://stats.oecd.org/, accessed 22 January 2018); Please note that total health expenditure (THE, per capita, constant prices, PPP) was divided by 1000 for the empirical analysis. *Access regulations*: based on MISSOC Comparative Tables Database, tables update for 1 July 2008 (http://www.missoc.org, accessed 22 January 2018) and WHO Health in Transition (HiT) country reports (see http://www.euro.who.int/countryinformation, accessed 22 January 2018), as well as previous research on access regulations and coding schemes [20,22]; Coding of access regulation is as follows: GP restriction is coded as 1 = choice restricted; 0 = no restriction/free choice; Access to specialist is coded as: 1 = GP referral; 2= skip and pay; 0 = free access to specialist; *Evaluation of native population*: average scores for native population calculated on the basis of the European Social Survey, rounds 1–7, standard weights were applied, scale ranges from 1 to 10; * If no information on GP (per 1000) was available (CH, DK, FI, HU, SK), information on the generalist medical practitioners (density per 1000 population) was used based on OECD database (http://stats.oecd.org/, accessed 22 January 2018); +Please note that the healthcare system of Belgium is coded as a skip and pay system as it offers increased reimbursement of health payments, if a referral from a GP is provided.
