# Supplementary material for: Beyond endogeneity in analyses of public opinion: Evaluations of healthcare by the foreign born across 24 European countries
Source: PLoS One. 2020 Jun 1;15(6):e0233835. doi: 10.1371/journal.pone.0233835 (PMC7263607; doi:10.1371/journal.pone.0233835)
Supplement: S4 Table — (PDF) [file pone.0233835.s004.pdf]

Table S4. Individual-Level characteristics, mean values/proportions

Variable	Mean Value, %
Evaluation of healthcare services (metric, scale: 0–10)	6.20
Length of stay (1 = < 6 year, 0 = other)	.18
Experienced discrimination (1 = yes, 0 = no)	.06
Gender (1 = female, 0 = male)	.56
Age in years (metric)	50.76
Self-reported health (metric, scale: -2 to 2, 0 = fair health)	.92
Education: (1 = low, 0 = other)	.28
Education: (1 = middle, 0 = other)	.34
Education: (1 = high, 0 = other)	.38
Subj. Income (1 = living comfortably, 0 = other)	.40
Subj. Income (1 = coping, 0 = other)	.41
Subj. Income (1 = (very) difficult, 0 = other)	.18
Employment (1 = paid work, 0 = other)	.54
Employment (1 = unemployed, 0 = other)	.05
Employment (1 = not in labour force, 0 = other)	.41
Year of survey (1 = 2002, 0 = other)	.18
Year of survey (1 = 2004, 0 = other)	.18
Year of survey (1 = 2006, 0 = other)	.14
Year of survey (1 = 2008, 0 = other)	.16
Year of survey (1 = 2010, 0 = other)	.11
Year of survey (1 = 2012, 0 = other)	.11
Year of survey (1 = 2014, 0 = other)	.12

Note: European Social Survey, rounds 1–7, sample population: foreign born respondents in Europe; table reports mean values/proportions on all individual-level characteristics of sample population
