# Supplementary material for: Beyond endogeneity in analyses of public opinion: Evaluations of healthcare by the foreign born across 24 European countries
Source: PLoS One. 2020 Jun 1;15(6):e0233835. doi: 10.1371/journal.pone.0233835 (PMC7263607; doi:10.1371/journal.pone.0233835)
Supplement: S5 Table — (PDF) [file pone.0233835.s005.pdf]

S5 Table. Effects of institutional healthcare characteristics on healthcare ratings – with varying cluster sizes at group level

Inst. Characteristics	Level of Effect	Cluster Size: min. 1				Cluster Size: min. 5				Cluster Size: min. 7			
		Model 1		Model 2		Model 1		Model 2		Model 1		Model 2	
		β	SE	β	SE	β	SE	β	SE	β	SE	β	SE
**Reference: Table 1**													
THE	Country level	.54***	.12	.51***	.12	.46***	.13	.44***	.13	.49***	.13	.46***	.12
	Group level	.09*	.04	.08$^+$	.05	.18**	.05	.16**	.05	.17**	.05	.14**	.05
	Cross-level			.02	.07			.04	.09			.04	.09
**Reference: Table 2**													
A Monetary Input													
PHE	Country level	.02	.02	.03	.02	.03	.03	.03	.02	.02	.02	.02	.02
	Group level	.01	.01	.01	.01	.01	.01	.01	.01	.02	.01	.02	.01
	Cross-level			.01	.01			.01	.01			.01	.01
PHE-gov.	Country level	-.00	.01	.00	.01	-.00	.01	.00	.01	-.00	.01	.00	.01
	Group level	-.01**	.00	-.01*	.00	-.01**	.00	-.01*	.00	-.01*	.00	-.01$^+$	.00
	Cross-level			-.00**	.00			.01**	.00			-.00*	.00
PHE-ins.	Country level	.00	.01	-.00	.01	.00	.01	.00	.01	.00	.01	.00	.01
	Group level	.01**	.00	.01**	.00	.01**	.00	.01**	.00	.01*	.00	.01*	.00
	Cross-level			.01**	.00			.01**	.00			.01**	.00
OOP	Country level	-.01	.02	-.01	.02	-.01	.02	-.01	.02	-.01	.02	-.01	.02
	Group level	-.02**	.01	-.02*	.01	-.02$^+$	.01	-.02	.01	-.02*	.01	-.02*	.01
	Cross-level			-.01	.01			-.01	.01			-.02$^+$	.01
B Real Input													
GP	Country level	1.16$^+$	.70	1.19*	.59	1.24$^+$	.70	1.30*	.55	1.14$^+$	.68	1.23*	.54
	Group level	.36**	.11	.27$^+$	.15	.31**	.10	.17	.13	.37***	.09	.21*	.10
	Cross-level			.29	.22			.43+	.22			.51$^+$	.28
Specialists	Country level	-.07	.39	-.13	.39	.01	.45	-.02	.46	.04	.44	.03	.46
	Group level	.06	.10	.05	.09	.01	.08	.02	.07	-.04	.09	-.01	.08
	Cross-level			.15	.18			.08	.20			.01	.21
H-Beds	Country level	.02	.11	-.03	.10	.03	.12	-.03	.10	.04	.12	-.02	.10
	Group level	.10**	.04	.08*	.04	.11**	.04	.10*	.04	.10*	.04	.08$^+$	.04
	Cross-level			.11***	.03			.12***	.03			.13***	.04
C Access Regulation													
Access - Specialists	Country level: referral by GP (0 = no)	.05	.43	.11	.41	-.04	.48	.06	.45	-.11	.49	-.02	.45
	Country level: skip & pay	-.25	.58	-.15	.55	-.32	.64	-.15	.62	-.30	.65	-.12	.63
	Group level: more freedom (0 = no change)	.27$^+$	.14	.32**	.12	.19	.17	.25*	.12	.19	.18	.26$^+$	.13
	Group level: less freedom	-.15	.10	.02	.12	-.35**	.10	-.19	.12	-.21*	.09	-.03	.11
	Cross-level: more freedom x recent mig.			-.29	.23			-.22	.26			-.28	.27
	Cross-level: less freedom x recent mig.			-.80***	.21			-.74**	.27			-.80**	.29
Access – GP	Country level: provider restriction (0 = no)	.53	.35	.55	.36	.74*	.35	.77*	.33	.53	.35	.55	.34
	Group level: more freedom (0 = no change)	.24*	.12	.17	.13	.33*	.14	.26$^+$	.14	.32*	.14	.26$^+$	.14
	Group level: less freedom	-.56***	.14	-.54***	.14	-.74***	.18	-.73***	.18	-.53**	.17	-.54***	.15
	Interaction: more freedom x recent mig.			.56***	.11			.59***	.13			.57***	.14
	Interaction: less freedom x recent mig.			-.03	.26			-.16	.27			-.05	.30
D Soft Institutions													
Native's Opinion	Country level	.67***	.12	.66***	.12	.65***	.12	.66***	.13	.64***	.12	.64***	.13
	Group level	.13*	.06	.14**	.05	.15*	.07	.15*	.06	.15*	.07	.14*	.06
	Cross-level			-.10	.09			-.11	.10			-.10	.09

Note: European Social Survey, round 1-7, sample population: foreign born respondents in Europe; multilevel analysis based on three levels: individuals/foreign born, migrant groups, countries of residence; sample sizes differ with the restriction on cluster size: cluster size with min. 1: individuals/foreign born (N= 6196), groups of foreign born (N = 347), countries of residence (N = 24); cluster size with min. 5: individuals/foreign born (N= 5831), groups of foreign born (N = 168), countries of residence (N = 24); cluster size with min. 7: individuals/foreign born (N= 5666), groups of foreign born (N = 138), countries of residence (N = 24); table reports unstandardized coefficients (β) and standard errors (SE); all analyses are based on Table 1 and control for experienced discrimination, length of stay in country of residence, demographic (sex, age) and socio-economic characteristics (education, income, employment status) and year of survey at the individual level, and the direct and interactional effects of the absolute and relative amount of total health expenditure (per capita, constant prices, PPP) at the country and group level (see Table 1); $^{+}$ $p < 0.10$, * $p < 0.05$, ** $p < 0.01$, *** $p < 0.001$ (two-tailed test); Abbreviations used in the table are as follows: THE: Total Health Expenditure (per capita, constant , PPP); PHE: Public Health Expenditure (% THE); PHE-gov.: Public Health Expenditure of Government Schemes (% THE); PHE-ins.: Public Health Expenditure of Compulsory Insurance Schemes (% THE); OOP: Out-of-Pocket Expenditure (% THE); GP: Density of GPs (per 1000); Specialists: Density of Specialists (per 1000); H-Beds: Density of Hospital Beds (per 1000); Access Specialists: Restricted Access to Specialist Care Services; Access – GP: Provider Restriction for Primary Care Services; Natives' Opinion: Natives' Opinion on Healthcare (mean values); Cross-level: Cross-level interaction with recent migration;
